# Supplementary figures and images for: The genetics and evolution of eye color in domestic pigeons (Columba livia)
Source: PLoS Genet. 2021 Aug 30;17(8):e1009770. doi: 10.1371/journal.pgen.1009770 (PMC8432899; doi:10.1371/journal.pgen.1009770)

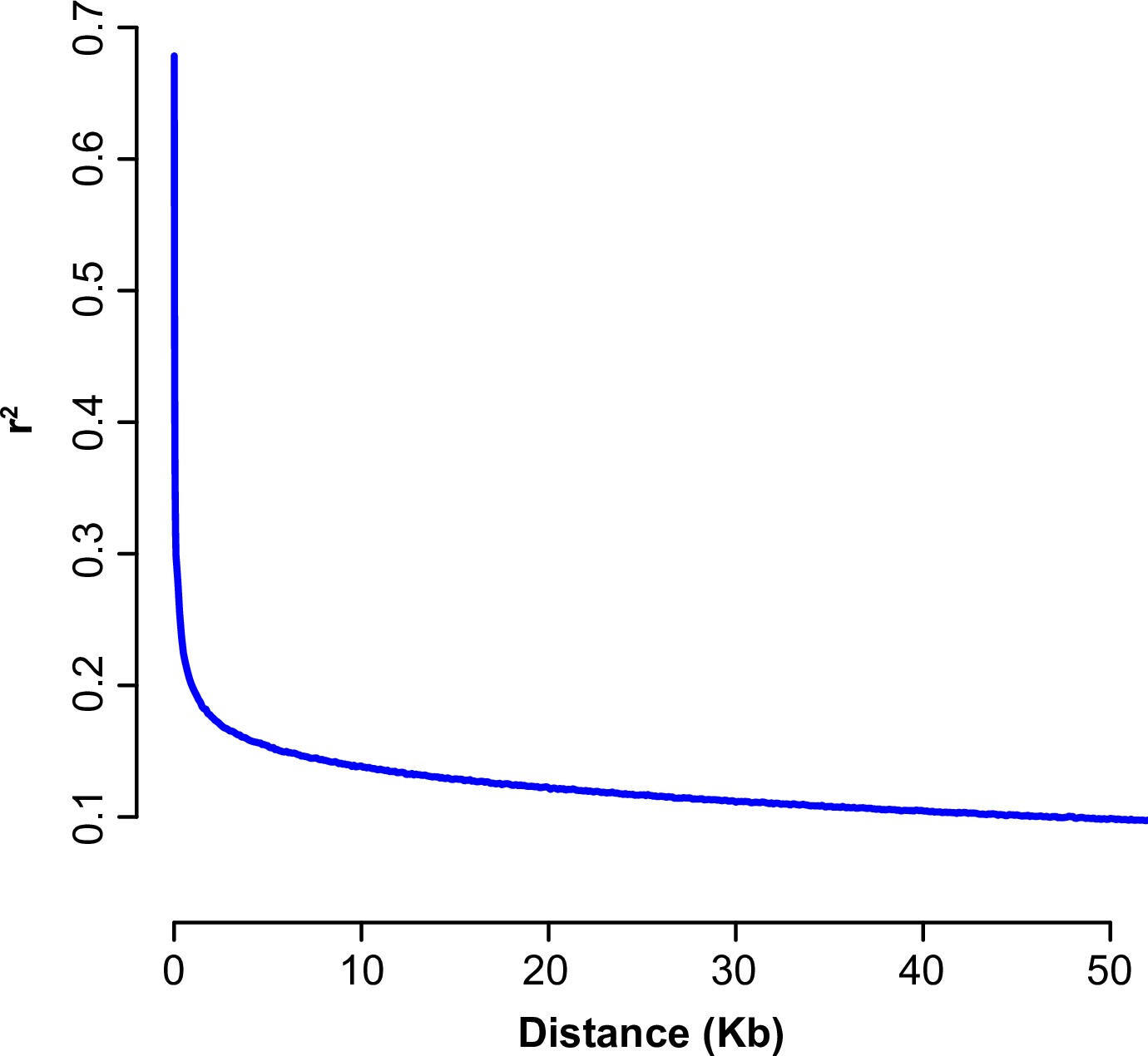

Supplement: S1 Fig — The LD decay curve is based on the mean correlation coefficient (r2) between common SNPs (minor allele frequency ≥ 0.1). The threshold for “useful LD” is set with r2 < 0.2 at distances beyond 0.9 Kb. Under this scenario, the pigeon genome shows a rapid LD decay suggesting that the genome-wide SNPs generated from the sample set are nearly or completely independent from each other, and hence are sufficient for association mapping in pigeons. (TIF) [file pgen.1009770.s001.tif]

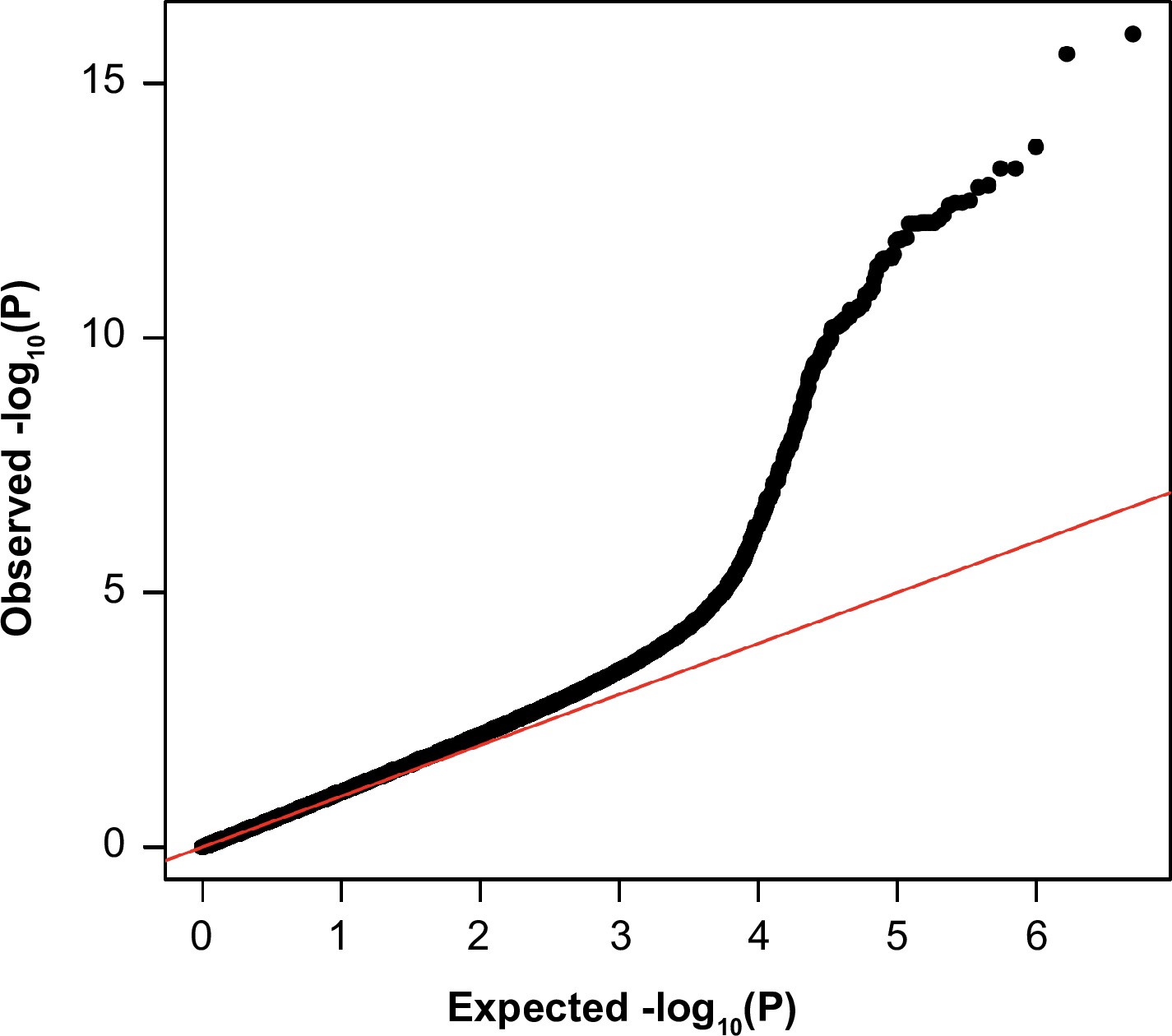

Supplement: S2 Fig — The observed versus expected quantiles of the genome-wide association P-value shown in Fig 2A. (TIF) [file pgen.1009770.s002.tif]

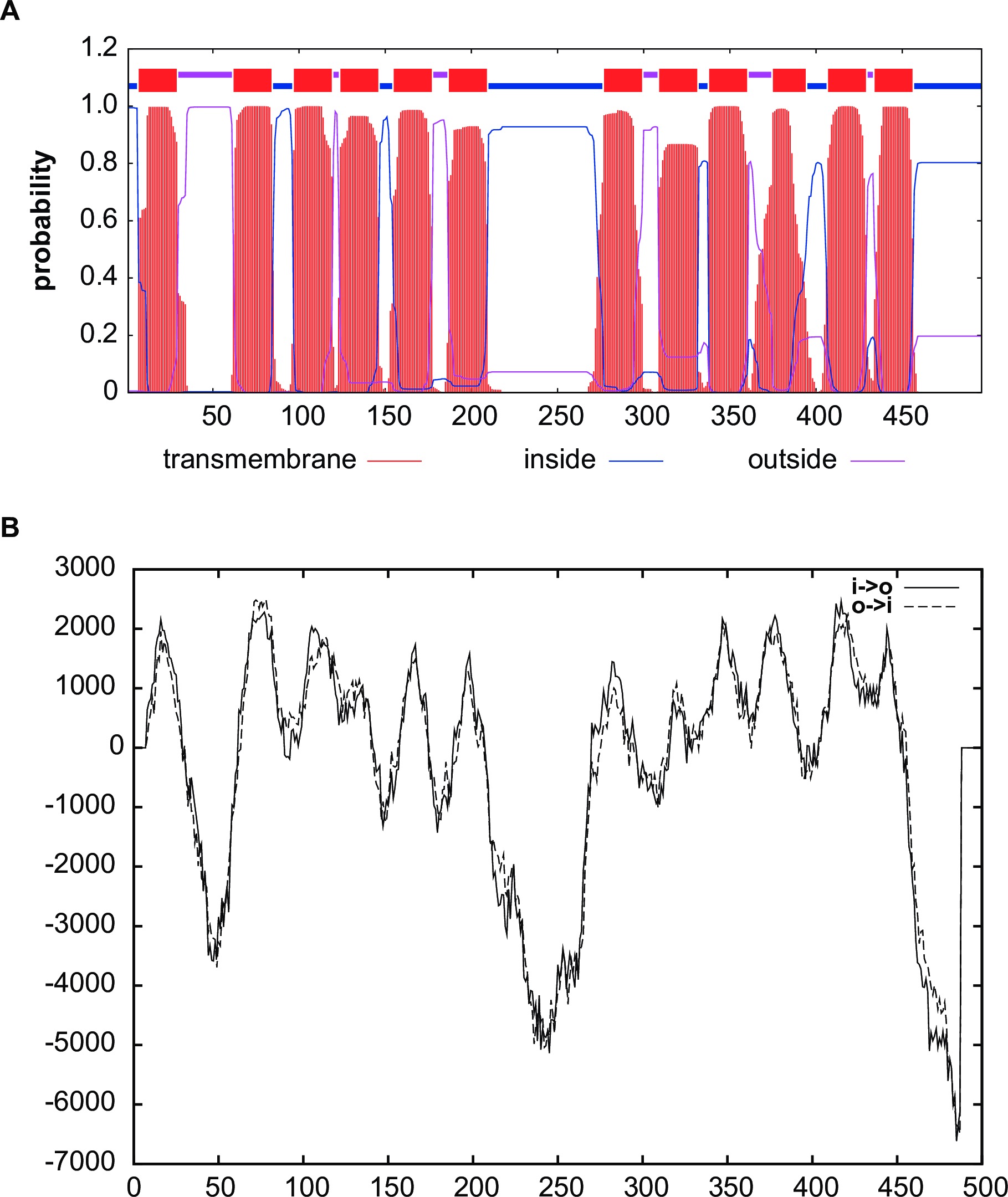

Supplement: S3 Fig — (A) TMHMM posterior probabilities of inside/outside/TM helix. The N-best prediction is displayed at the top where transmembrane regions are shown in red boxes. (B) Result output from TMpred server. The predicted transmembrane helices with scores above 500 are considered significant. The solid and dashed line indicates inside-to-outside and outside-to-inside transmembrane helices, respectively. (TIF) [file pgen.1009770.s003.tif]

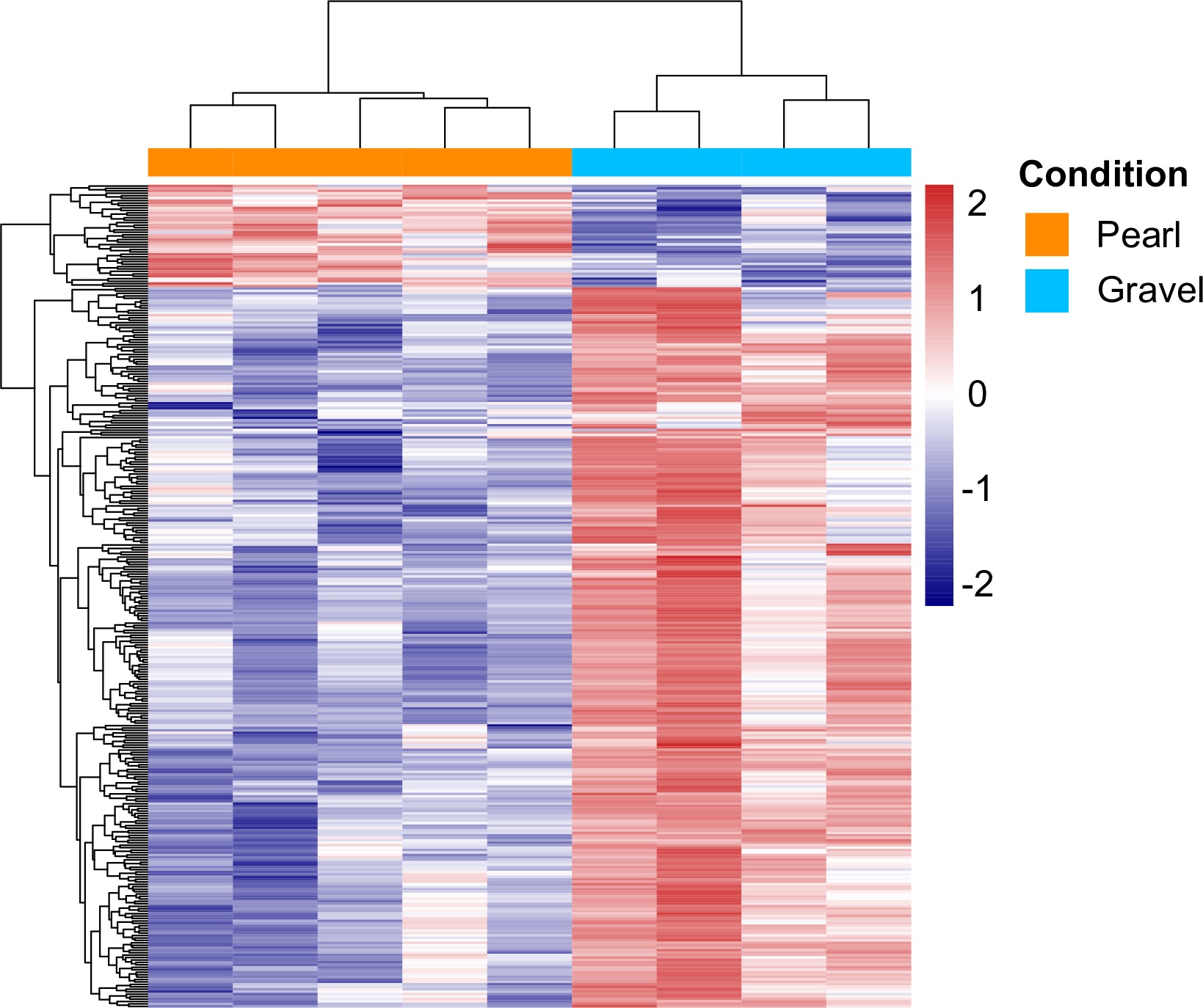

Supplement: S4 Fig — The heatmap, hierarchically clustered into two groups of genes, recapitulates a total of 337 differentially expressed genes (DEGs), among which 295 and 42 genes were specifically upregulated in gravel (N = 4) and pearl (N = 5) irises, respectively. Columns are individual samples and rows indicate individual genes. The level of expression is color-coded from DESeq normalized counts, with red representing the higher level of expression, blue the lower level. (TIF) [file pgen.1009770.s004.tif]

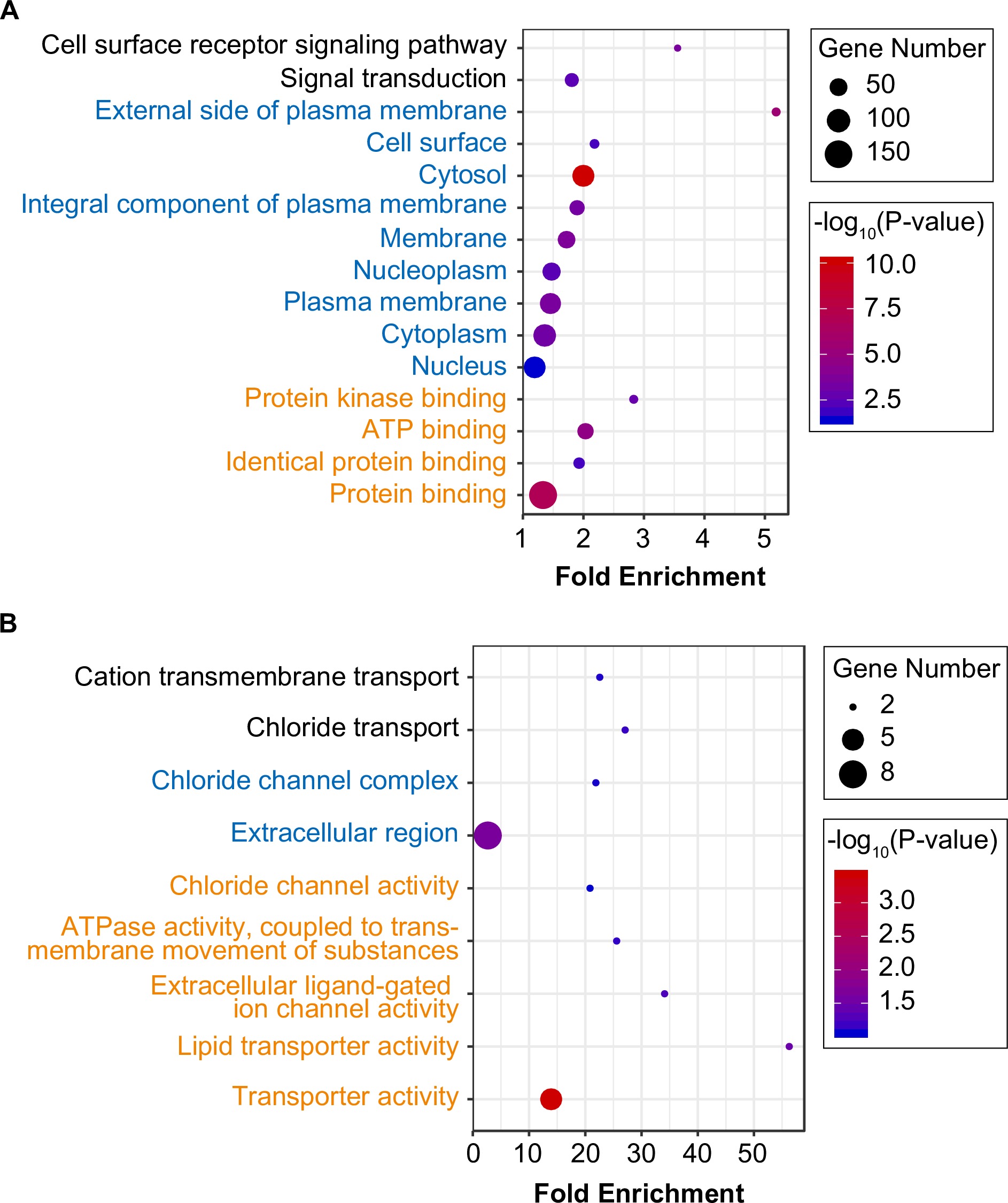

Supplement: S5 Fig — (A) GO enrichment of 295 DEGs upregulated in gravel iris. (B) GO enrichment of 42 DEGs upregulated in pearl iris. The bubble diagrams show the degree of enrichment of Gene Ontology (GO) terms in three categories. The orange, blue, and black represent molecular function (MF), cellular component (CC), and biology process (BP) categories, respectively. Each bubble indicates a GO term, and the size of bubbles is proportional to the number of genes annotated to the GO term. P-value is represented by the color map. (TIF) [file pgen.1009770.s005.tif]

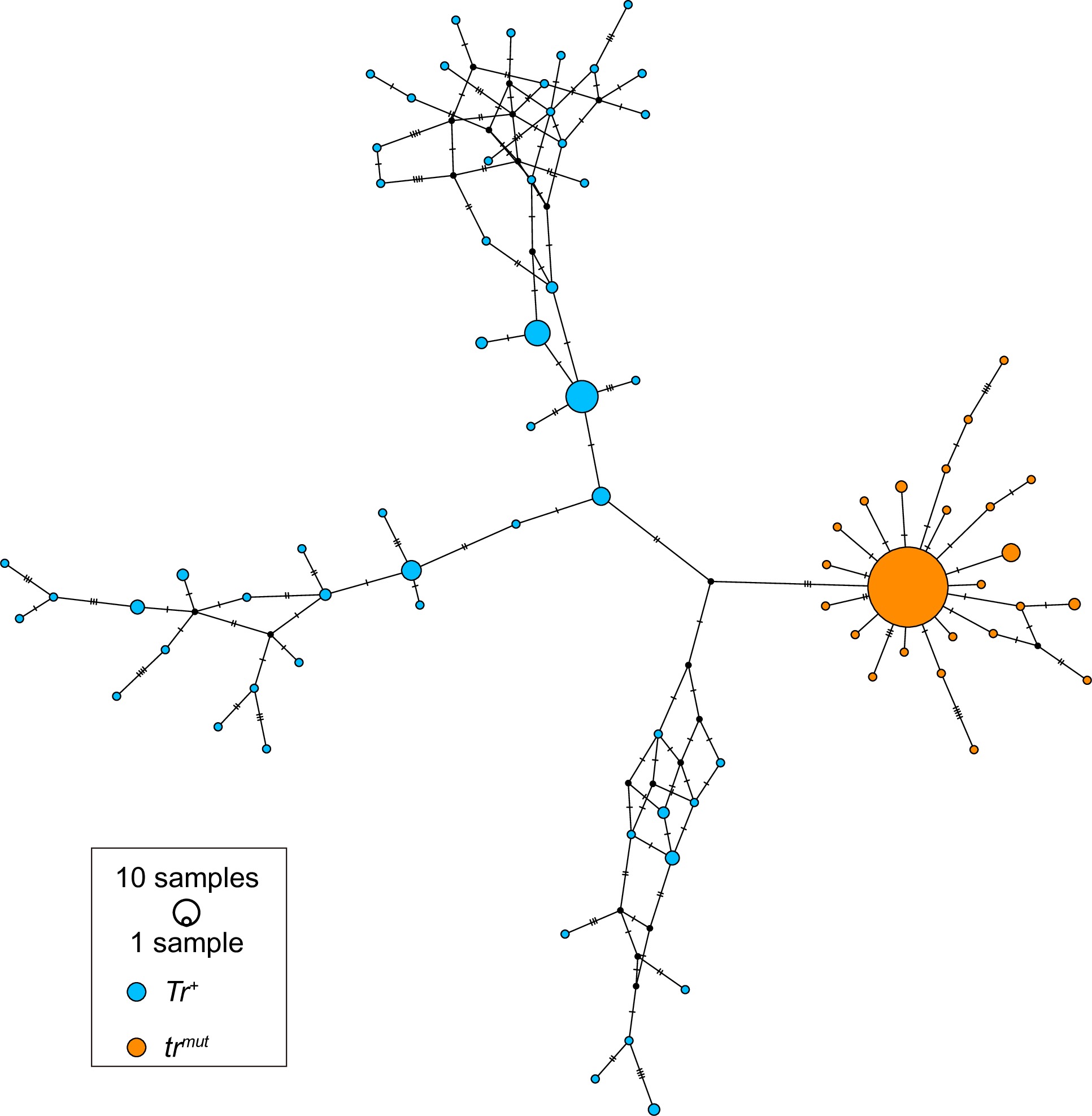

Supplement: S6 Fig — The haplotype network was generated from 9 Kb nonrecombining Tr region from 139 domestic pigeons (35 fancy pigeons, 2 feral pigeons, and 102 racing pigeons). The mutations are shown by hatch marks. The orange and blue circles represent trmut and Tr+ haplotypes, respectively. (TIF) [file pgen.1009770.s006.tif]

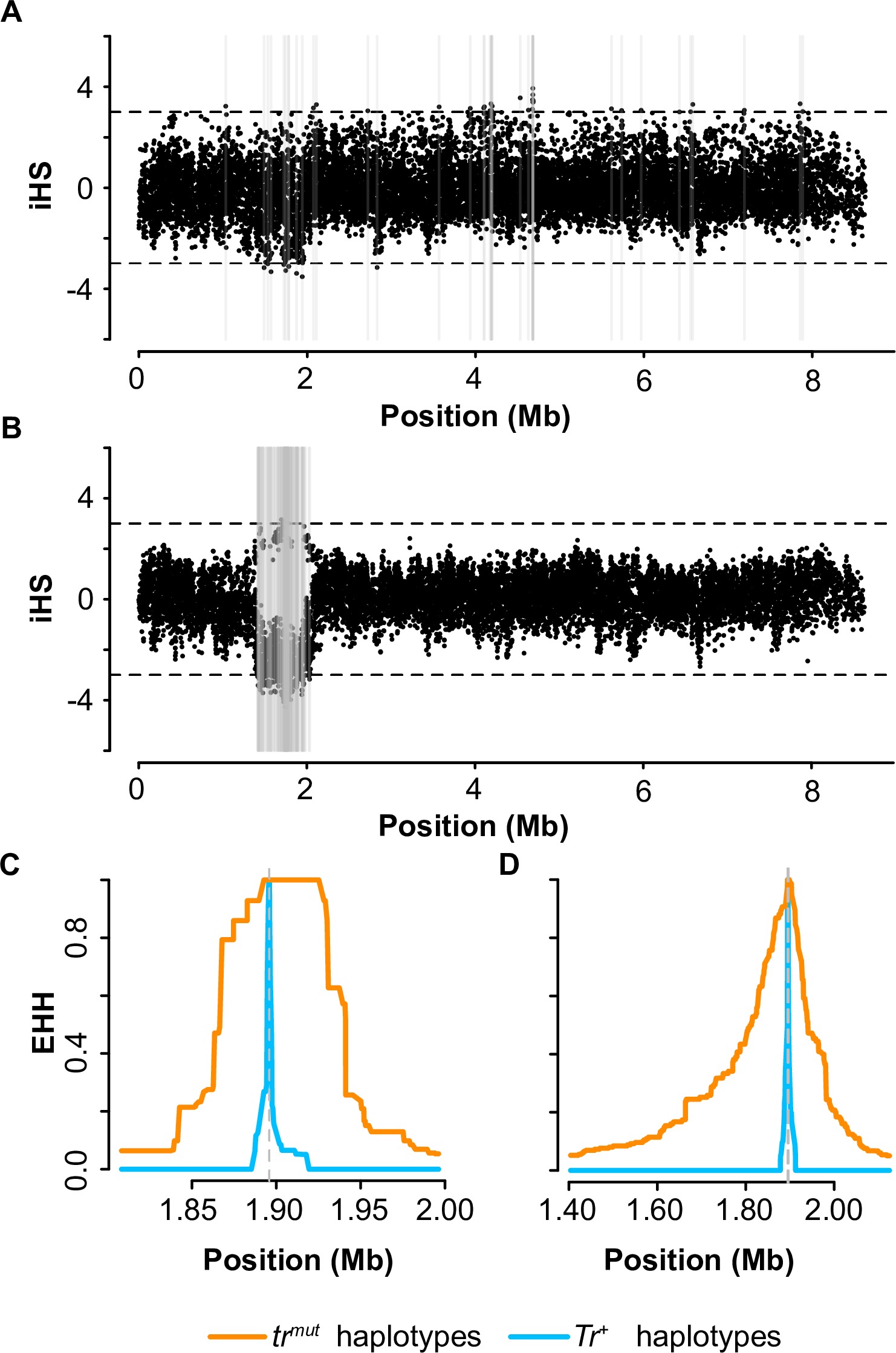

Supplement: S7 Fig — The integrated haplotype score (iHS) was calculated for scaffold AKCR02000030.1 in fancy pigeons (A) and racing pigeons (B). The gray lines represent the significance level of absolute iHS scores of 3 or greater. The extended haplotype homozygosity (EHH) decay across the Tr locus region is showed for fancy pigeons (C) and racing pigeons (D). (TIF) [file pgen.1009770.s007.tif]

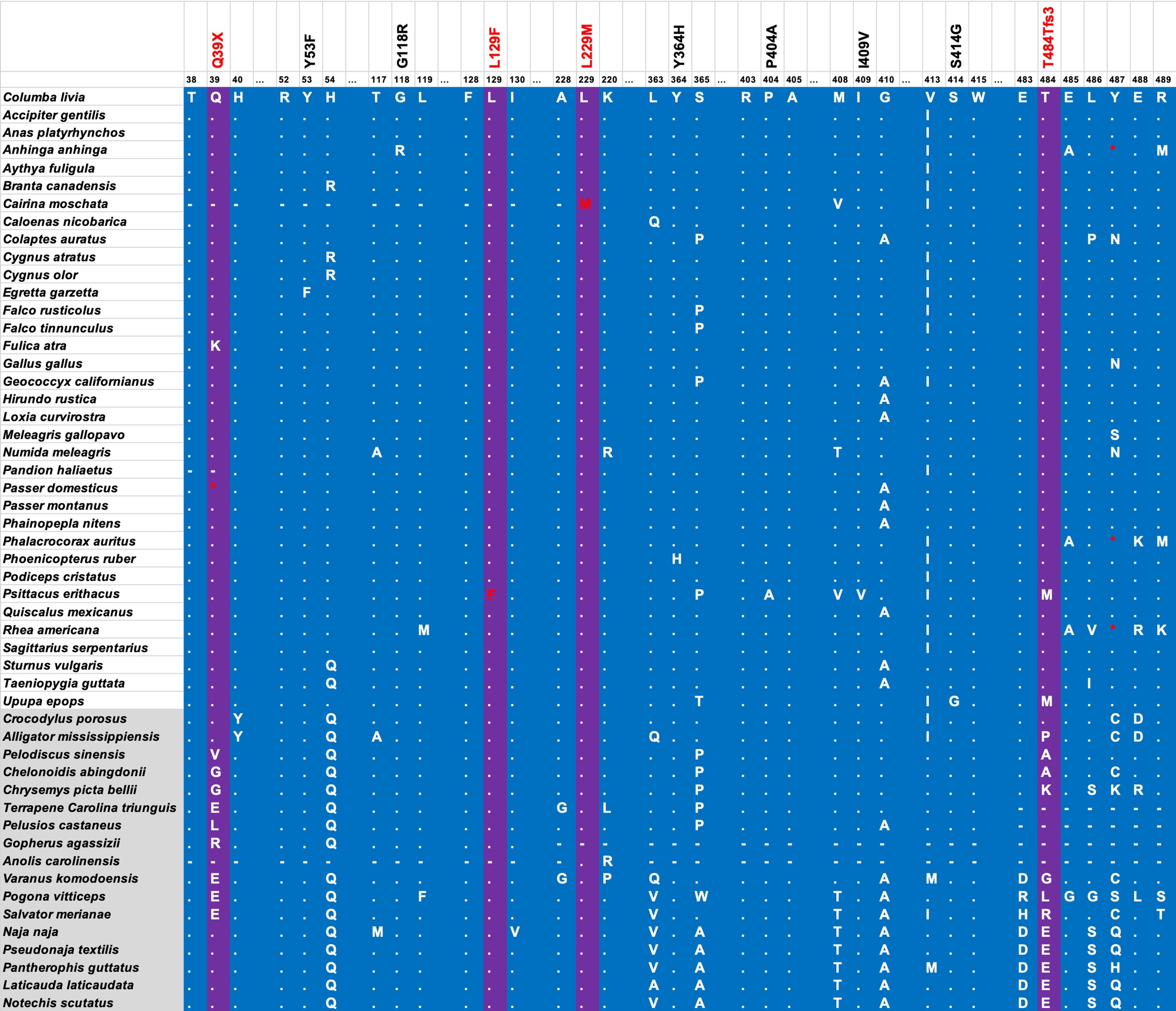

Supplement: S8 Fig — A total of 52 coding sequences of 35 Aves, 2 Crocodilia, 6 Testudines, and 9 Squamata were aligned. The partial alignment of amino acid sequences of avian SLC2A11B with conserved species-specific mutations is shown. The conserved species-specific nonsense, missense and frame-shifting mutations are labeled on the top of the table, and the deleterious mutations are marked in red. (TIF) [file pgen.1009770.s008.tif]
