# Supplementary material for: The genetics and evolution of eye color in domestic pigeons (Columba livia)
Source: PLoS Genet. 2021 Aug 30;17(8):e1009770. doi: 10.1371/journal.pgen.1009770 (PMC8432899; doi:10.1371/journal.pgen.1009770)
Supplement: Attachment — Submitted filename: Si_Xu_et_al_review.docx [file pgen.1009770.s017.docx]

In this manuscript by Si and Xu et al, the authors map the Mendelian pearl eye locus in domestic pigeons (which causes complete loss of pterin pigmentation in the iris) to a single locus containing the gene SLC2A11B. They find that presence of a premature stop codon in this gene correlates almost perfectly with the trait, and follow up with RNA-sequencing data from irises of pearl eye versus wild-type birds to show that this genetic model is the most likely one. In addition, the authors estimated the age of the causative allele, and found signatures of artificial selection at that locus.

Artificial selection for various traits in pigeons, many of them Mendelian, has long been a favorite topic for biologists. I enjoyed reading this work and I believe others will too. In addition to linking phenotype to genotype, it also raises interesting questions about how cell types such as xanthophores and irridophores have evolved.

I would be happy to consider a revised version of this manuscript that addresses the following three Main Points:

**Main Points:**

1) Linkage group information should be added to Table S3. For the purposes of ruling out the alternative hypothesis (although unlikely) that the pearl eye phenotype is caused by a regulatory variant, it is important to check if any of the differentially expressed genes reside on the same linkage group as the GWAS peak. This can be done using Table S6 from Holt et al (<https://doi.org/10.1534/g3.117.300443>), which is attached.

2) Some of the statements concerning the mechanism of *SLC2A11B* in causing pearl eye are overly speculative, and rely mostly on data from zebrafish rather than pigeons, for instance the heading on p6: "**SLC2A11B W49X affects stromal pigment cell differentiation in pigeons**". If the authors had shown that there were many fewer stromal pigment cells in pearl irises, or that these cells were somehow malformed, for example by in situ hybridization against a known marker gene for that cell type, this statement would be justified. Certainly the fact that *CSF1R* is downregulated in pearl irises is consistent with this model. But there are other plausible explanations for the observed bulk expression data that don't involve a differentiation defect. Therefore, I suggest changing the wording to something like: *"Genes with known roles in stromal cell differentiation are downregulated in W49X birds"*, or something to that effect. There are statements on p9 and p13 that should be similarly amended.

3) I'm not convinced that the phenotype data presented in the section "**SLC2A11B is involved in avian iris color variation"** are of high enough quality to support many of the claims made there. It is not clear how the data in the "Iris color" column of Table S5 were collected, but I would assume it was from photographs. The problem with this method is that one cannot always tell from presence or absence of yellow color whether pteridine pigments are present or not (some species have yellow irises due to carotenoids). Has it been shown somewhere that birds with brown irises never have pteridine pigments? As far as I can tell, there have been only two studies that analyzed iris pteridine pigments in a wide sample of species, and the latter did not look at any brown-eyed species:

Oehme, H. 1969. Vergleichende Untersuchungen uber die Farbung der Vogeliris. Biologische Zentralblatt 88: 3-35.

Oliphant, L.W. 1987. Pteridines and purines as major pigments of the avian iris. Pigment Cell Research 1: 129-131.

Given this uncertainty regarding the phenotypes, I suggest limiting this section to species where the pigment phenotype is definitely known, such as cormorants. If some of the species from the Oehme paper have sequenced genomes, that would be worth including, but it seems to be a difficult paper to get a copy of (I have only seen summaries of it in other published work).

**Minor Points:**

1) p4: *"Here, we investigated the genetic basis and origin of bright irises (gravel and pearl eyes) in domestic pigeons."* This should read "origin of pearl irises in domestic pigeons". Finding a gene required for bright irises is not the same thing as elucidating the origin of bright irises.
2) p5: *"(GWAS) based on whole-genome resequencing was performed in domestic pigeons (racing homers) to identify the genomic region responsible for the bright iris phenotype."* Should read "responsible for the pearl eye phenotype." See minor point #1.
3) p7: There should be p-values in the text for the differential expression results.
4) p14: *"In pigeons, SLC2A11B exhibits a tissue-specific expression pattern".* Since *SLC2A11B* is also expressed in muscle and brain, this is not quite accurate. This should be rephrased to something like "among epithelial tissues, the expression of SLC2A11B is specific to the iris" or something to that effect.
5) p22: *"Individuals homozygous for the tr^mut^ allele were set as the reference panel."* Shouldn't individuals homozygous for *Tr+* should be the reference panel, since *tr^mut^* is the selected allele?
