# Supplementary material for: The genetics and evolution of eye color in domestic pigeons (Columba livia)
Source: PLoS Genet. 2021 Aug 30;17(8):e1009770. doi: 10.1371/journal.pgen.1009770 (PMC8432899; doi:10.1371/journal.pgen.1009770)
Supplement: Attachment — Submitted filename: response_to_comments_20210617.docx [file pgen.1009770.s019.docx]

**Point-by-Point Responses to Reviewers’ Comments (Responses in Bold fonts)**

Reviewer #1:	In this manuscript by Si and Xu et al, the authors map the Mendelian pearl eye locus in domestic pigeons (which causes complete loss of pterin pigmentation in the iris) to a single locus containing the gene SLC2A11B. They find that presence of a premature stop codon in this gene correlates almost perfectly with the trait, and follow up with RNA-sequencing data from irises of pearl eye versus wild-type birds to show that this genetic model is the most likely one. In addition, the authors estimated the age of the causative allele, and found signatures of artificial selection at that locus.

Artificial selection for various traits in pigeons, many of them Mendelian, has long been a favorite topic for biologists. I enjoyed reading this work and I believe others will too. In addition to linking phenotype to genotype, it also raises interesting questions about how cell types such as xanthophores and irridophores have evolved.

I would be happy to consider a revised version of this manuscript that addresses the following three Main Points:

**R1: We appreciate the reviewer for the positive recognition of our manuscript.**

Main Points:

1) Linkage group information should be added to Table S3. For the purposes of ruling out the alternative hypothesis (although unlikely) that the pearl eye phenotype is caused by a regulatory variant, it is important to check if any of the differentially expressed genes reside on the same linkage group as the GWAS peak. This can be done using Table S6 from Holt et al (<https://doi.org/10.1534/g3.117.300443>), which is attached.

**R2: We added the linkage group information in Table S3 based on Holt et al. 2018. There are six differentially expressed genes (*IGLL1*, *GGT1*, *TBX3*, *KNTC1*, *PRC1*, and *A306_00008692*) located in the same linkage group as the GWAS peak, and two of them (*GGT1* and *TBX3*) are pigmentation genes involving in melanocyte activities in mammals, therefore we cannot completely rule out the possibility of regulatory variants. Nevertheless, since none of the six genes is known to have any function associated with xanthophore or leucophore, we consider this alternative hypothesis highly unlikely to explain the pigmentation change in pearl-eyed pigeons. We added a paragraph to describe this result in lines 233-240, and we also added a sentence to describe the source of linkage group information in lines 651-652.**

**Lines 233-240:“In addition, located in the same linkage group with *SLC2A11B* we found six DEGs (*IGLL1*, *GGT1*, *TBX3*, *KNTC1*, *PRC1*, and *A306_00008692*; S3 Table), among which *GGT1* and *TBX3* are involved in the regulation of melanocyte in mammals [39, 40]. Although we cannot completely rule out the possibility that potential regulatory variants within the GWAS candidate interval may cause pearl eyes, this alternative hypothesis is highly unlikely, as none of the six DEGs is known to have any function associated with xanthophore or leucophore, and hence could not explain the pigmentation change in pearl-eyed pigeons.”**

**Lines 651-652: “The linkage group information for each DEG was extracted from a previous study [34].”**

2) Some of the statements concerning the mechanism of SLC2A11B in causing pearl eye are overly speculative, and rely mostly on data from zebrafish rather than pigeons, for instance the heading on p6: "SLC2A11B W49X affects stromal pigment cell differentiation in pigeons". If the authors had shown that there were many fewer stromal pigment cells in pearl irises, or that these cells were somehow malformed, for example by in situ hybridization against a known marker gene for that cell type, this statement would be justified. Certainly the fact that CSF1R is downregulated in pearl irises is consistent with this model. But there are other plausible explanations for the observed bulk expression data that don't involve a differentiation defect. Therefore, I suggest changing the wording to something like: "Genes with known roles in stromal cell differentiation are downregulated in W49X birds", or something to that effect. There are statements on p9 and p13 that should be similarly amended.

**R3: We revised the heading into “Genes with known functions in xanthophore/leucophore pigmentation are downregulated in SLC2A11B W49X pigeon iris” in lines 199-200, and we also rephrased the statements throughout the section in lines 309-310 and 424-425, to avoid over-speculation.**

3) I'm not convinced that the phenotype data presented in the section "SLC2A11B is involved in avian iris color variation" are of high enough quality to support many of the claims made there. It is not clear how the data in the "Iris color" column of Table S5 were collected, but I would assume it was from photographs. The problem with this method is that one cannot always tell from presence or absence of yellow color whether pteridine pigments are present or not (some species have yellow irises due to carotenoids). Has it been shown somewhere that birds with brown irises never have pteridine pigments? As far as I can tell, there have been only two studies that analyzed iris pteridine pigments in a wide sample of species, and the latter did not look at any brown-eyed species:

Oehme, H. 1969. Vergleichende Untersuchungen uber die Farbung der Vogeliris. Biologische Zentralblatt 88: 3-35.

Oliphant, L.W. 1987. Pteridines and purines as major pigments of the avian iris. Pigment Cell Research 1: 129-131.

Given this uncertainty regarding the phenotypes, I suggest limiting this section to species where the pigment phenotype is definitely known, such as cormorants. If some of the species from the Oehme paper have sequenced genomes, that would be worth including, but it seems to be a difficult paper to get a copy of (I have only seen summaries of it in other published work).

**R4: Thank you for the insightful comment and the reference suggestion. We have retrieved a copy of the Oehme 1969 paper from the journal’s original hard copy subscribed by our Peking University library (quite amazing). In the previous version of our manuscript, the avian iris phenotype was indeed obtained from photographs. We agree with the reviewer that based on the iris coloration alone, we cannot accurately identify the pigments, as Oliphant 1987 showed both pteridines and carotenoids can cause similar iris color. In this revision, we followed the review’s suggestion and analyzed the SLC2A11B in 34 avian species whose iris pigmentation information (from the papers of Oehme 1969, Oliphant 1987 and Oliphant et al. 1992) and genome data are both available. We found 4 mutations that might affect the SLC2A11B function in 6 bird species, with which Q39X in house sparrow, T484Tfs3 in the double-crested cormorant and greater rhea, and L229M in Muscovy duck supported our hypothesis that deficiency of SLC2A11B might be associated with the absence of pteridines in avian irises. The new results were described in lines 309-338 under a new heading “SLC2A11B is associated with avian iris color variation”, and were discussed in lines 452-470.**

Minor Points:

1) p4: "Here, we investigated the genetic basis and origin of bright irises (gravel and pearl eyes) in domestic pigeons." This should read "origin of pearl irises in domestic pigeons". Finding a gene required for bright irises is not the same thing as elucidating the origin of bright irises.

**R5: Revised in line 123.**

**“Here, we investigated the genetic basis of pearl irises in domestic pigeons.”**

2) p5: "(GWAS) based on whole-genome resequencing was performed in domestic pigeons (racing homers) to identify the genomic region responsible for the bright iris phenotype." Should read "responsible for the pearl eye phenotype." See minor point #1.

**R6: Revised in line 137.**

**“……to identify the genomic region responsible for the pearl eye phenotype.”**

3) p7: There should be p-values in the text for the differential expression results.

**R7: We included a statistical test to display the relative expression level of SLC2A11B in multiple tissues. We rewrote the related results and added p-value where appropriate in the text in lines 202-213.**

4) p14: "In pigeons, SLC2A11B exhibits a tissue-specific expression pattern". Since SLC2A11B is also expressed in muscle and brain, this is not quite accurate. This should be rephrased to something like "among epithelial tissues, the expression of SLC2A11B is specific to the iris" or something to that effect.

**R8: We revised the sentence in lines 485-486.**

**“Among the pigeon epithelial tissues, the expression of SLC2A11B is specific to the iris.”**

5) p22: "Individuals homozygous for the tr^mut^ allele were set as the reference panel." Shouldn't individuals homozygous for Tr+ should be the reference panel, since tr^mut^ is the selected allele?

**R9: Yes, the *Tr+* homozygous individuals should be the reference panel. We corrected the typo in line 727.**

Reviewer #2: In this study the authors identify genetic variants potentially involved in the pearl eyed phenotype of domestic pigeons. After successfully demonstrating a causative variant, the authors search for associations with eye color in other birds and also test the hypothesis that this gene has undergone relaxed selection in birds. I found the paper to be well written with many interesting observations carefully unpacked. I read the paper with consideration of another similar publication in the same journal by Andrade et al, which also identified the same causative variants. I applaud the authors of the current study for their phylogenetic comparative analyses, which goes beyond what was done in the other manuscript, and is a compelling addition.

**R10: We thank the reviewer for the positive comments on our manuscript, especially the recognition of the additional value of our paper comparing to Andrade et al. that was just published in PLoS Gen. identifying the same variant.**

I enjoyed the analyses conducted on the gene of interest with other avian species. I expect these types of analyses will become more common with the many genomes becoming available and I see this as a good example of how fruitful these comparative analyses are going to be. On the other hand, I did not find the analyses conducted on the history of the gene outside of birds to be as easy to follow. That birds have retained some sort of functional expression of SLC2A11B in the eye, but not other tissue, is certainly interesting. However, I found it difficult to 1) postulate that this gene is degenerate when it is associated with eye color across numerous birds, 2) that a relaxation of selection on this gene is due to the evolution of feathers in birds when many non-avian dinosaurs had feathers, 3) link the data in the paper about eye coloration to a broad phylogenetic analysis that is largely based on dermal pigments, and 4) fully embrace the results when TYR shows inconsistent result with what is written in the paper. I expand on this somewhat in my comments below, but I personally would gain the same value from this manuscript if the RELAX analysis were not included and the focus was more specifically on the role of SLC2A11B in birds in general. An enlightening discussion could still be made more broadly about the history of chromatophores in the reptilian radiation.

**R11: Thanks for the comment. We have addressed the issue regarding RELAX analysis in the responses.**

The version of the ms I received did not have line numbers, so I do my best to be specific with pages and paragraphs in my comments below:

**R12: We apologized for the inconvenience. Line numbers have been added in this revision.**

Abstract: “elucidate” is vague as to what will be made clearer. Reflecting on what is done in the study, I suggest “explore” as a potential alternative here.

**R13: We revised “elucidate” into “explore” in line 27.**

Page 3, paragraph 2: last sentence – birds are vertebrates so this should possibly read “other vertebrates” or be expanded.

**R14: We revised “vertebrates” into “poikilothermic vertebrates” in line 78.**

Paragraph 3 –Intraspecific variation in wild birds is often associated with age (e.g. https://doi.org/10.1111/j.1474-919X.2006.00630.x) and sex (examples include accipiters, bushtits, others). Suffice to say intraspecific variation is not limited to domestic species.

**R15: The sentence was revised into “Intraspecific eye color variation is also common and often associated with age and sex in wild birds [8, 10-12].” in lines 86-87.**

Page 4, 2nd paragraph: A citation is needed for the types of pigment cells in other avian species

**R16: The citations have been added in lines 115-116: “Similar stromal pigment cells and/or developmental color changes were found in the irises of other avian species [8, 12, 32, 33].”**

Figure 1 should have a photo credit

**R17: Photo credit “J. -O. Gao” has been added to Figure 1 caption in line 121.**

Page 4, last paragraph: “aves coloration” should be “avian coloration”

**R18: Done in line 131.**

Page 6 “The iris pigmentation in pearl-eyed pigeons resembles that of SLC2A11B loss of function in fish, supporting W49X of SLC2A11B as the causal mutation responsible for the pearl eye color” In what way is the iris pigmentation similar, in the pigments themselves or in appearance?

**R19: The iris color change from gravel to pearl eye in pigeon is similar to the skin color change from wild type to SLC2A11B deficiency in fish. To avoid potential confusion, we revised the sentence into “The pigmentation change of the iris stromal pigment cells in pearl-eyed pigeons resembles the leucophore color switch from yellow to white in medaka fish larva with SLC2A11B loss-of-function [35], thus supporting W49X of SLC2A11B as the causal mutation responsible for the pearl eye color” in lines 181-185.**

Page 6: “...mutation was further validated in..” By which method were they validated?

**R20: To validate the W49X mutation, we amplified exon 3 of SLC2A11B by PCR and sequenced the amplicons in 292 pigeons. We revised the sentence into “This nonsense mutation was further validated in 146 gravel-eyed and 146 pearl-eyed individuals by PCR and Sanger sequencing” in lines 185-186.**

Page 6: “However, five pearl-eyed pigeons were heterozygous for the SLC2A11B p.W49X variant (Fig. 3B) with no other mutations found in the exons of SLC2A11B.” For ease of readership, I suggest that there is a mention of the argument described in discussion that these could have been mis-phenotyped.

**R21: Thanks for the suggestion. We have added a sentence accordingly: “Such phenotype-genotype incompatibility in these five outliers could have been due to mis-phenotyping of young pigeons or observer error.” in lines 191-193.**

Page 7/Figure 4A: Be more specific about high or low expression (it is only meaningful in relation to other tissue), I suggest including a statistical test that will allow you to state that SLC2A11B expression is higher in brain, iris, retina, and muscle than other tissue.

**R22: We included a statistical test and compared the SLC2A11B expression of skin with that of other tissues to indicate the relative expression level. The related results were rewritten with more details in lines 202-213. Also, please refer to R7 above.**

Page 7: One alternative (although not mutually exclusive) explanation for the results in Andrade et al is that there were two other genes also on the same linkage group that were found to be differentially expressed (CHEK2 and TRAFD1). The potential for other cis-regulatory variants located in the region of differentiation detected here is unlikely, but possible. It is worth checking these two in the RNAseq data as it will support, or perhaps suggest against, this possibility of other regulatory variants.

**R23: We thank both reviewers for raising the issue. We have addressed this issue accordingly. Please refer to R2 above. We also noticed that the six differentially expressed genes we identified within the same linkage group do not overlap with the two reported by Andrade et al. We are aware that in our study 5 pearl vs. 4 gravel iris samples were used for RNAseq, while the corresponding sample set in Andrade et al. was 2 vs. 4. We used more than double the sample size for the pearl-eye pigeons. The sample size contrast and individual variance may explain the difference between the two studies.**

Page 7: “SLC2A11B was slightly downregulated, by 1.2- fold, in the pearl iris (Fig. 4B), likely caused by nonsense-mediated RNA decay.” I found it disingenuous to not point out that SLC2A11B is not significantly differentially expressed in the RNAseq analysis in this place (it is only in the supplemental that I can make this connection). What is the explanation for the significant difference in qPCR, but not RNAseq? It is difficult to speculate based on Figure 4C, which would benefit from including an overlay of the data points on the boxplots.

**R24: SLC2A11B was not significantly differentially expressed in RNAseq analysis, but we did observe a downregulation trend in pearl-eyed pigeons, and the downregulation of SLC2A11b in pearl individual was further validated by qPCR. Discordance of expression results between RNAseq and qPCR is not rare, maybe because RNAseq does not always provide representative read distribution across the full length RNA. Usually, the DEGs detected by RNAseq need to be validated by qPCR. Here we rephrased the sentence into “*SLC2A11B* was not significantly differentially expressed, but a downregulation trend of *SLC2A11B*, likely caused by nonsense-mediated RNA decay, was evident in pearl-eyed individuals (Fig 4B), which was further confirmed by qPCR (Fig 4C).” in lines 216-219. Figure 4C was modified as suggested, with an overlay of data points on the boxplots.**

Page 8: “Such widespread prevalence across pigeon breeds is consistent with a relatively longstanding history of pearl eye color in domestic pigeons.” This could be interpreted in one of two ways – that the genotype is old, or that the pearl eye phenotype is widespread across breeds. My suggestion is that if it is the latter, to put a number on how many of these 36 breeds are known to have the pearl eyed phenotype (or if it is all breeds, write that).

**R25: In this revised manuscript we used genome data from 35 breeds, instead of 36. Genome of the English pouter (SRS346884) was discarded, due to the poor genotype quality at the *Tr* mutation locus. In this version, 45 pigeons from 35 breeds were analyzed. Review of the breed standards information resulted in presence of the iris color phenotype was obtained for 31 of the 35 breeds, within which 17 breeds have pearl eye phenotype. The iris color information for each breed was added in Table S4. And we revised the sentence into “Such widespread SLC2A11B W49X allele frequency is consistent with the phenotypic prevalence across pigeons, in that at least 17 of the 35 breeds are independently documented to carry the pearl eye trait (S4 Table)” in lines 256-258.**

Page 8: As this is the first mention of C. rupestris, I suggest you make a small note that this species is considered sister to all others in the dataset.

**R26: We revised the sentence as suggested in line 262.**

Page 8: Figure 5 caption does not match the text. The text reads that a NJ tree was made from the SLC2A11B genic sequence, but the caption reads that it was the 9kb region of association. I do not know which is shown in the figure.

**R27: We revised the text into “To further investigate the origin of the SLC2A11B W49X mutation, a neighbor-joining phylogenetic tree was reconstructed based upon the 9 kb nonrecombining *Tr* region from 139 domestic pigeons (35 fancy pigeons, 2 feral pigeons, and 102 racing homers) and one *C. rupestris* that is considered sister taxa to all domestic pigeons (Fig 5A)” in lines 259-263.**

Page 8: What method was used to date the TMRCA?

**R28: We used the software startmrca to date the TMRCA. The details were described in Methods in lines 720-736.**

Page 9: The section title would be more appropriately written as “SLC2A11B is associated with avian iris color variation”

**R29: We thank both reviewers for raising the issue. We have addressed this issue accordingly. Please also refer to R4 above. We revised the heading as suggested in line 308.**

**Following the other reviewer’s suggesting, this section has been re-analyzed based on bird species with documented iris pigments information. We analyzed SLC2A11B in 34 avian species whose iris pigmentation information and genome data are both available. We found 4 mutations that might affect the SLC2A11B function in 6 bird species, within which Q39X in house sparrow, T484Tfs3 in the double-crested cormorant and greater rhea, and L229M in Muscovy duck supported our hypothesis that deficiency of SLC2A11B might be associated with the absence of pteridines in avian irises. The new results were described in lines 309-338, and were discussed in lines 452-470. Please also refer to R4.**

Page 11: “On the other hand, the absence of relaxed selection was consistently supported by all three schemes for TYR, a key melanogenesis gene that is expected to be evolutionarily conserved and under similar selective pressure across all vertebrates.” This is at odds with the table in Figure 7 – here k <1 and p = 5e-9 for TYR in the clade comparison.

**R30: To avoid confusion, we revised the sentence into “In contrast, no relaxed selection was consistently supported for any clade by more than one schemes for *TYR*, a key melanogenesis gene that is expected to be evolutionarily conserved and under similar selective pressure across all vertebrates” in lines 374-377.**

Page 12: “as most likely responsible” would be appropriate in this case

**R31: Revised in line 386.**

Page 13: small typo: “it might be more appropriate to refer *to* pigeon iris stromal cells”

**R32: Revised in line 422.**

Page 13: “which indicated that CSF1R acts downstream of SLC2A11B in the regulatory network” this is somewhat speculative, I suggest some caveats such as “which is consistent with the possibility that CSF1R is downstream...” or similar

**R33: We revised the sentence into “……which is consistent with the scenario that *CSF1R* is downstream of *SLC2A11B* in the regulatory network of xanthophore/leucophore differentiation.” in lines 424-426.**

Page 14: “One landmark evolutionary transition setting the stage for the divergence of avians from their dinosaur ancestors was the development of feathery coverage that concealed their skin pigmentation.” This is an oversimplification given that there are now numerous fossilized non-avian dinosaurs suggesting that some dinosaurs had feathers.

**R34: Thanks for the comments regarding the feathered non-avian dinosaurs. The sentence has been revised to “One landmark evolutionary transition setting the stage for the origin of birds was the development of feathery coverage that concealed their skin pigmentation in their feathered, non-avian dinosaur ancestors.” in lines 471-473.**

It is also an oversimplification to state that the only important pigments in birds are related to feather color, as many species have colorful bare parts (e.g. see this review on bare parts signaling <https://doi.org/10.1642/AUK-16-136.1>).

**R35: Here we meant the epidermal melanocyte is the only pigment cell remained in birds, except for the stromal pigment cells in the iris which remain the full potential for developing into all types of chromatophores. In order to avoid confusion, we revised the sentence into “In modern birds, the epidermal melanocyte is the only pigment cell distributed throughout the body and plays an important role in integument pigmentation.” in lines 477-479.**

Although this tells a compelling story arc, the inconsistency in results relating to TYR in the analysis, and the uncertainty of SLC2A11B’s role in other coloration types in birds, would give me some uncertainty in describing the history of this gene in this way.

**R36: Since relaxation of selection is the alternative hypothesis in the RELAX test and is what we want to rigorously detect, we reckon it is reasonable to claim relaxation only when test on all three schemes show significance. By this criterion, only SLC2A11B experienced relaxation. TYR only showed significance under the clade scheme, but no sign of relaxation when the scheme of either the internal branches or the basal branch of the avian clade is considered. We have modified the description of test results in the main text to be consistent with Figure 7 as mentioned above, and avoid over-speculation of SLC2A11B’s role in other coloration types in birds in Discussion. Please also refer to R30 above.**

I also find it difficult to reconcile the widespread phylogenetic signal of SLC2A11B’s role in eye coloration across birds (suggesting at least some degree of purifying selection is taking place) and the hypothesis that this gene has undergone evolutionary demise.

**R37: While we have found relaxation of selection on SLC2A11B across birds, the conceptual interpretation of this result is not equivalent to the evolutionary demise of this gene, because a weaker selection may still exist for SLC2A11B, e.g. due to pleiotropic effects suggested by its expression in organs other than the iris, or potential adaptive significance in generating iris color, as we mentioned in discussion. In this sense, stochastic loss-of-function mutation for SLC2A11B may not happen at a rate high enough to cause complete loss of phylogenetic signal across the avian clade, while we did observe multiple smaller-scale cases in species like house sparrow and Muscovy duck. Hence, we reckon our proposed scenario of SLC2A11B evolution is not in conflict with the observation of its phylogenetic existence pattern in birds.**

“dinosaur ancestors” could be rephrased as non-avian dinosaur.

**R38: Thanks for the comment. We have screened through the text and made corrections accordingly.**

Page 15: The IACUC # should be appropriate here

**R39: IACUC# LSC-LuoSJ-2 is provided in Methods, line 527.**

Page 17: It is not clear which versions of GATK were used for what steps during SNP calling

**R40: GATK v4.0.2.1 was used for SNP calling. We made a correction in line 565.**

Page 19: What metric was used to determine significantly differentially expressed genes in DESeq2? E.g. a p-value cutoff, or p-value + log fold change cut off.

**R41: We added the threshold for DEGs in lines 648-649.**

Page 20: This should have a citation or perhaps expand on the methods used “Experimental data were manually analyzed in a normalized expression comparative Ct (-2∆∆Ct) model.”

**R42: A citation was added for referring the comparative analysis of qPCR in line 667.**

Supplemental information:

Figure S1: “Despite the rapid LD decay indicated that the clumps of SNPs were nearly or completely independent from each other, domestic pigeons were well suited for association-mapping methods considering on the sufficiently genome- wide marker density generated by SNPs calling.” This should be rephrased for clarity.

**R43: The legend has been rewritten:**

**“Figure S1. The linkage disequilibrium (LD) decay in the pigeon genome. The LD decay curve is based on the mean correlation coefficient (r^2^) between common SNPs (minor allele frequency ≥ 0.1). The threshold for “useful LD” is set with r^2^ < 0.2 at distances beyond 0.9 Kb. Under this scenario, the pigeon genome shows a rapid LD decay suggesting that the genome-wide SNPs generated from the sample set are nearly or completely independent from each other, and hence are sufficient for association mapping in pigeons.”**

I believe some of the genomes in Table S5 (e.g. Anna’s Hummingbird) should be attributed to this preprint from VGP: https://doi.org/10.1101/2020.05.22.110833

**R44: We only included in the analysis the genomes of 34 avian species whose iris pigment information is documented, following the suggestion of Reviewer #1. Table S5 has been revised accordingly. All references for the avian genomes used in the study were included in Table S5. In addition, SLC2A11B variations at the evolutionarily conserved sites were added in Table S5.**
