# Supplementary material for: The genetics and evolution of eye color in domestic pigeons (Columba livia)
Source: PLoS Genet. 2021 Aug 30;17(8):e1009770. doi: 10.1371/journal.pgen.1009770 (PMC8432899; doi:10.1371/journal.pgen.1009770)
Supplement: Attachment — Submitted filename: Response_to_Comments_20210723.docx [file pgen.1009770.s020.docx]

**Point-by-Point Responses to Reviewers’ Comments (Responses in Bold fonts)**

Reviewer #1: The changes to the sections "Genes with known functions in xanthophore/leucophore pigmentation are downregulated in SLC2A11B W49X pigeon iris" and "SLC2A11B is associated with avian iris color variation" address all the major points I raised in my original review. It was a pleasure to review.

**R: We appreciate the reviewer’s effort in improving our manuscript.**

Reviewer #2: I was reviewer #2 on the original submission of the manuscript. I appreciate the responses to my comments on the earlier version, and feel that the manuscript has improved in this revision.

I spent quite a bit of time revisiting the comments regarding the RELAX analysis, and re-reading the original manuscript for the software (Wertheim et al 2015). I appreciate that the authors gave my comments some thought, which has helped guide me on how the authors are interpreting the data regarding evolutionary demise. One point I remain unclear on is why the test is only considered significant when there are all three schemes supporting a model of relaxed selection. There seems to be compelling evidence that TYR is under relaxed selection in birds and the same can be said for the relaxed selection in squamata and the testudines, which also suggest relaxation under some schemes (table S6).

Some further justification, when these schemes are introduced in the results, would help clarify the interpretation that SLC2A11B has undergone relaxed selection. For example, is there a reason the authors believe one scheme over another? I imagine the internal nodes may have lower sampling variance as there is more data available than on single branches (as seems to be indicated in the RELAX manuscript), but in that case it is not clear why setting up different schemes is necessary vs. reporting values of k at nodes and terminal branches.

I appreciate the compelling premise that dermal chromatophores have undergone demise, a relaxation of selection has occurred for chromatophore-linked genes such as this, and then possibly a chromatophores have been repurposed in the eyes of birds. However, as there is some speculation involved here, it is key is that the molecular data backs this up as the most likely explanation, or at least there is clear justification for the way the data is presented and interpreted.

**R: We thank the reviewer for rigorously checking the logic of the test scheme design. We have added conceptual justification of the design in the main text (Page 12 Line 354):**

**“As the test involves modeling and inference of multiple mutational and selection parameters, information provided by sequence states of a single gene (636 codon sites for *SLC2A11B* and 572 codon sites for *TYR*) on a few branches may be limited. To avoid spurious significance and to strengthen data inference, we set three different schemes of test/reference branch designation, … Consistent results across three different schemes, or, the same conclusions based on information from different branches, would be most convincing.”**

**We also discussed why the significant test result for TYR in clade scheme is at least not related to the scenario we want to test (Page 13 Line 383):**

**“Specifically, test for avian *TYR* is only significant under the clade scheme while not significant when testing the basal or internal branches, indicating the relaxation, if any, did not arise until the relatively recent diversification of birds. Hence, this is not likely to be related to the initial avian divergence from reptile ancestors. ”**

**We hope in this sense, we justify to a satisfactory extent the reason for using three different test schemes and claiming relaxation only when all schemes lead to the same conclusion.**
